# Loss of AKR1C1 is a good prognostic factor in advanced NPC cases and increases chemosensitivity to cisplatin in NPC cells

**DOI:** 10.1111/jcmm.15291

**Published:** 2020-04-19

**Authors:** Chen Zhou, Guowen Shen, Fan Yang, Jingling Duan, Zhen Wu, Mingqing Yang, Yi Liu, Xueli Du, Xiaoling Zhang, Shengjun Xiao

**Affiliations:** ^1^ Department of Pathology The Second Affiliated Hospital Guilin Medical University Guilin China; ^2^ Department of Pathology Fudan University Shanghai Cancer Center Shanghai China; ^3^ Xiangya Medical College of South Central University Changsha China; ^4^ Department of Physiology Faculty of Basic Medical Science Guilin Medical University Guilin China

**Keywords:** AKR1C1, chemosensitivity, cisplatin, nasopharyngeal carcinoma

## Abstract

Cisplatin resistance is one of the main obstacles in the treatment of advanced nasopharyngeal carcinoma (NPC). AKR1C1 is a member of the Aldo‐keto reductase superfamily (AKRs), which converts aldehydes and ketones to their corresponding alcohols and has been reported to be involved in chemotherapeutic resistance of multiple drugs. The expression and function of AKR1C1 in NPC have not been reported until now. The aim of this research was to investigate the expression of AKR1C1 and it is role in cisplatin resistance in NPC. AKR1C1 protein expression was detected by immunohistochemistry in human NPC tissues and by Western blot assays in NPC and immortalized nasopharyngeal epithelial cells. The effects of AKR1C1 knock‐down by siRNA on proliferation, migration and invasion in NPC cells were evaluated by CCK8, wound healing and transwell assays. To evaluate the effects of AKR1C1 silencing on cisplatin sensitivity in NPC cells, CCK8 assays were used to detect cell proliferation, flow cytometry was used to detect cell cycle distribution, and flow cytometry and DAPI staining were used to detect cell apoptosis. AKR1C1 down‐regulation was associated with advanced clinicopathological characters such as larger tumor size, more lymphatic nodes involvement, with metastasis and later clinical stages, while AKR1C1 down‐regulation was a good prognostic factor for overall survival (OS) in NPC patients. In vitro study showed that AKR1C1 was not directly involved in the malignant biological behaviours such as proliferation, cell cycle progression and migration of NPC cells, whereas AKR1C1 knock‐down could enhance cisplatin sensitivity of NPC cells. These results suggest that AKR1C1 is a potential marker for predicting cisplatin response and could serve as a molecular target to increase cisplatin sensitivity in NPC.

## 1 INTRODUCTION

Nasopharyngeal carcinoma (NPC) is a endemic malignant tumour which has a high incidence in Southern China.^1^ In recent years, improved radiotherapy techniques have given satisfactory outcome in early stage of NPC.^2^ But most of patients were diagnosed as locally advanced NPC at the first diagnosis and their 5‐year survival rate was still only about 30%‐80%.^3^, ^4^, ^5^ As indicated by the National Comprehensive Cancer Network (NCCN) Guidelines, locoregionally advanced disease requires cisplatin‐based concurrent chemoradiotherapy.^1^

Cisplatin‐based regimen has been proposed as the optimal protocol by a meta‐analysis of eight randomized trials including 1753 patients.^6^ Unfortunately, cisplatin resistance occurs in some NPC cases and becomes a major obstacle of chemotherapy success for NPC.^7^ Thus, understanding the mechanism of cisplatin resistance in NPC may enable the development of new strategies to overcome chemoresistance and improve clinical outcome in locally advanced NPC cases.

Human 20‐keto reductase family 1 member C1 (AKR1C1) is a member of the aldehyde ketone reductase superfamily (AKRs).^8^ AKR family members catalyse the conversion of aldehydes and ketones to their corresponding alcohols.^9^ Their substrates include endogenous and xenobiotic non‐steroidal carbonyl compounds.^10^ Moreover, chemotherapeutic drugs containing carbonyl can be converted to inactivated reductive metabolite, leading to the chemotherapy resistance.^10^ Actually, cumulative data indicated that AKR1C1 plays an important role in chemotherapy resistance in several cancers.^11^, ^12^, ^13^, ^14^ Thus, targeting AKR family members provide a novel therapeutic strategy for overcoming chemoresistance in malignant tumours.

Up‐regulation of the AKR1C1 gene in multiple cancer cells was reported to be associated with resistance against several anticancer agents including cisplatin.^11^, ^12^, ^13^, ^14^ However, the expression and role of AKR1C1 in nasopharyngeal carcinoma has not been reported so far. In the present study, we found that AKR1C1 down‐regulated in advanced NPC tissues, but down‐regulated AKR1C1 was a good prognostic factor for overall survival (OS) in NPC patients. In vitro study indicated that AKR1C1 did not directly contribute to the malignant biological behaviours and knock‐down of AKR1C1 by siRNA increased the cisplatin sensitivity in NPC cells. Hence, AKR1C1‐targeted strategy may be a novel therapeutic candidate for overcome cisplatin resistance in NPC patients.

## 2 MATERIALS AND METHODS

### 2.1 Ethical approval

All procedures performed in studies involving human subjects met the ethical standards of the Institutional Review Board (IRB) of the Second Affiliated Hospital of Guilin Medical College (Guilin, China), and the Helsinki Declaration of 1964 and its subsequent amendments or similar ethical standards. The cells used for the study were approved by the IRB of the Second Affiliated Hospital of Guilin Medical College.

### 2.2 Patients and samples

Patients and samples in this study were shared with one of our previous studies, and their information was previously fully described.^15^

### 2.3 Immunohistochemical staining and microscopic analysis

Immunohistochemical assays were previously fully described.^15^ The dilution of anti‐AKR1C1 antibody (sc‐166297, SANTA CRUZ BIOTECHNOLOGY, USA) was 1:100. The secondary antibody (goat anti‐mouse) (Cat. No. KIT‐5001; MXB Biotechnologies) was ready to use. IHC scores were evaluated by two pathologists in our department. Staining intensity was classified as 0 (negative); 1 (weak); 2 (moderate); and 3 (strong). The scores 0 and 1 were defined as low, and the scores 2 and 3 were defined as high.^15^

### 2.4 Cell culture

Human NPC cell lines (namely CNE1, HK1‐EBV, CNE2, SUNE1, HONE1, 5‐8F and S18) and NP69, SXSW‐1489 and HNEpC cells were kindly provided by Prof. Dong Xiao (Institute of Cancer Research, Southern Medical University). All cells were cultured in RPMI‐1640 medium (Gibco) supplemented with 10% FBS (Gibco), at 37˚C in a humidified atmosphere with 5% CO_2_. All experiments were conducted on cells in the logarithmic growth stage.

### 2.5 AKR1C1‐siRNA transfection

The CNE1 and CNE2 cells were seeded in six‐well culture plates and cultured until the NPC cells reached 60%‐70% confluency. Si‐AKR1C1 and si‐ctrl (RiboBio) were transfected into the CNE1 and CNE2 cell lines by Lipofectamine 3000 (Invitrogen) according to the manufacturer's protocol. After transfection for 48 hours, AKR1C1 expression levels were checked by Western blot assays. Successfully transfected cells were used for subsequent experiments.

### 2.6 Western blotting assay

Total proteins in cells were lysed by RIPA lysate buffer (Beyotime Inc.) including benzosulfonyl fluoride (Beyotime Inc.) and protease inhibitor cocktail (Beyotime Inc.). SDS‐PAGE was conducted to isolate 20 μg of protein and transfer it to PVDF membrane (Millipore Inc., USA). The protein concentrations were measured with a BCA Protein Assay Kit (Beyotime Biotechnology). After blocked with 5% non‐fat milk for 2 hours at room temperature, the blotted membranes were incubated with AKR1C1 antibody (1:1000; Santa Cruz Biotechnology) overnight at 4˚C. The following day, HRP IgG secondary antibody (1:5000; ZSGB‐BIO) was incubated for 1 hours. Finally, blots were detected by ECL substrate (Beyotime Inc.), and Image J software was used to analysis the expression of protein.

### 2.7 Cisplatin treatment

To obtain an appropriate concentration of cisplatin for subsequent chemotherapeutic sensitivity assays, different concentrations (0.2, 0.4, 0.8, 1.6, 4.0 μg/mL) of cisplatin (QiLu Pharmaceutical Co) were used to determine the IC50 in NPC cells. In brief, CNE1 and CNE2 (4000 cells/well) were plated on 96‐well plates (Costar) with 5 wells/group and were cultured overnight. Then, the cells were treated with cisplatin for 48 hours and CCK8 assays were employed to detect cell growth. According to the IC50 (Figure S3), 0.5 μg/mL of cisplatin was selected for subsequent chemotoxicity assays. To detect the sensitized effects of AKR1C1 knock‐down on cisplatin (DDP) treatment in NPC cells, CNE1 and CNE2 were all divided into four groups, which including si‐ctrl, si‐AKR1C1, si‐ctrl + DDP and si‐AKR1C1 + DDP. After transfected with siRNA overnight, CNE1 and CNE2 cells were treated with DDP for 48 hours and then submitted for CCK8 assays.

### 2.8 CCK8 assay

CNE1 cells (4000 cells/plate) and CNE2 cells (4000 cells/plate) were grown in 96‐well plates (Costar). After the cells were cultured overnight, they were transfected with siRNA (RiboBio). Then, the cells were cultured for 24‐96 hours. The subsequent CCK8 assays were previously fully described.^15^

### 2.9 Wound healing assay

After the cells were transfected with siRNA, cell migratory ability was evaluated by wound healing assays. Wound healing assays were previously fully described.^15^ The wound healing distance was documented at 48 and 72 hours.

### 2.10 Transwell migration and invasion assays

Transwell assays were previously fully described.^15^ In this study, 2 × 10^4^ cells/well (migration assay) or 4 × 10^4^ cells/well (invasion assay) in 200 μl serum‐free RPMI 1640 were seeded on top of the filter. After 24 hours (migration assay) or 48 hours (invasion assay), the cells were fixed, stained and then calculated under a microscope.

### 2.11 Flow cytometry‐based cell cycle

After treated with cisplatin for 48 hours, each group of cells were washed with PBS for 3 times and fixed with 70% cold ethanol at 4°C overnight. Then, the cells were washed, centrifuged, resuspended and then stained with 200 μL PI/RNase Staining Buffer (BD, Biosciences). The cell cycle was measured using a FACScan flow cytometer (BD Biosciences) and analysed by ModFit software (BD Biosciences).

### 2.12 Flow cytometry‐based apoptosis

After treated with cisplatin for 48 hours, each group of cells was washed with PBS for 3 times and then harvested and stained with annexin V‐APC and 7AAD (RiboBio) according to the manual of Cell Apoptosis Kit. Apoptosis was immediately detected by flow cytometry after 15 minutes of incubation in the dark at room temperature.

### 2.13 DAPI staining

After treated with cisplatin for 48 hours, the cells were fixed with 4% paraformaldehyde for 10 minutes, permeabilized with 0.5% Triton X‐100 (ZSGB‐BIO) for 15 minutes and then counterstained with DAPI (Solarbio) 5 minutes. Finally, the cell slides were gently removed and sealed, and the images were observed under a fluorescence microscope.

## 3 RESULTS

### 3.1 AKR1C1 expression decreased in NPC tissues and frequently lost in NPC cells

**Figure 1:**
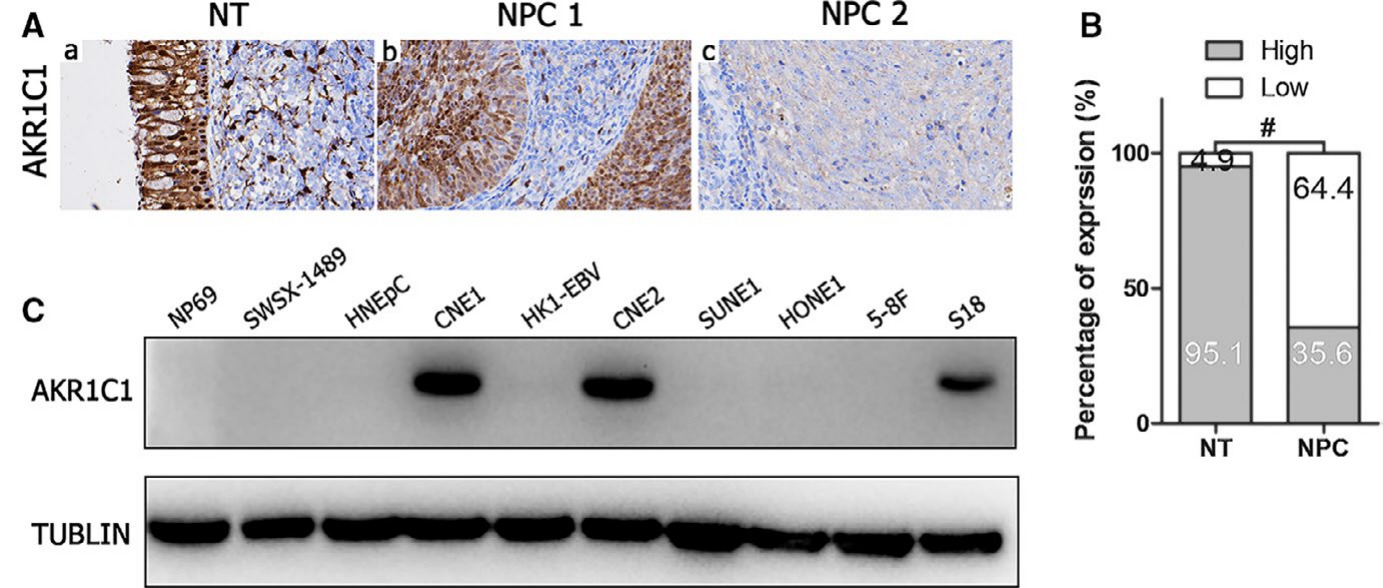
AKR1C1 expression decreased in NPC tissues and frequently lost in NPC cells. (A) AKR1C1 expression in non‐cancerous nasopharyngeal biopsies and NPC tissues based on IHC. a: High expression of AKR1C1 in non‐cancerous nasopharyngeal epithelia; b: high expression of AKR1C1 in NPC biopsies; c: low expression of AKR1C1 in NPC biopsies. The brown staining indicates AKR1C1 immunoreactivity. (B) AKR1C1 expression was significantly lower in the NPC biopsies than in the non‐cancerous nasopharyngeal biopsies. (C) Expression levels of AKR1C1 in the indicated immortalized nasopharyngeal epithelial cells and NPC cells were examined by Western blotting. Abbreviations: NT, non‐cancerous nasopharyngeal epithelia; NPC, nasopharyngeal carcinoma; AKR1C1, Human 20‐keto reductase family 1 member C1; IHC, immunohistochemical staining

To determine AKR1C1 expression levels in NPC tissues, 177 NPC tissues and 61 non‐cancerous epithelial tissues were detected by IHC. The IHC staining results revealed that AKR1C1 protein significantly down‐regulated in NPC specimens (Figure 1A and B and Table 1). When AKR1C1 expression evaluated by Western blotting in NPC cell lines (namely CNE1, HK1‐EBV, CNE2, SUNE1, HONE1, 5‐8F and S18) and immortalized nasopharyngeal epithelial cells (NP69, SWSX‐1489 and HNEpC), only three NPC cells (CNE1, CNE2 and S18) expressed AKR1C1 (Figure 1C). AKR1C1 strongly stained in normal ciliated columnar epithelia of nasopharyngeal tissue while lost in immortalized nasopharyngeal epithelial cells, the underlined mechanism needs to be elucidated. We found that AKR1C1 significantly down‐regulated in squamous metaplastic epithelia of nasopharyngeal by IHC (Figure S1 and Table S1). So the phenotype changes of immortalized nasopharyngeal epithelial cells in vitro still need to be identified. Anyway, the data above showed that AKR1C1 expression decreased in NPC tissues and frequently lost in NPC cells, which implied that loss of AKR1C1 was a key molecular event in the development of NPC.

**TABLE 1:** Expression of AKR1C1 in 61 non‐cancerous epithelial tissues and 177 NPC tissues

Variables	n	AKR1C1 expression (n, %)		χ^2^	*P*
		High	Low		
Non‐cancerous epithelial tissues	61	58 (95.08)	3 (4.92)	64.236	<.001
NPC	177	63(35.59)	114 (64.41)		

### 3.2 Loss of AKR1C1 was associated with advanced TNM stage while was a good prognostic factor in NPC patients

**Figure 2:**
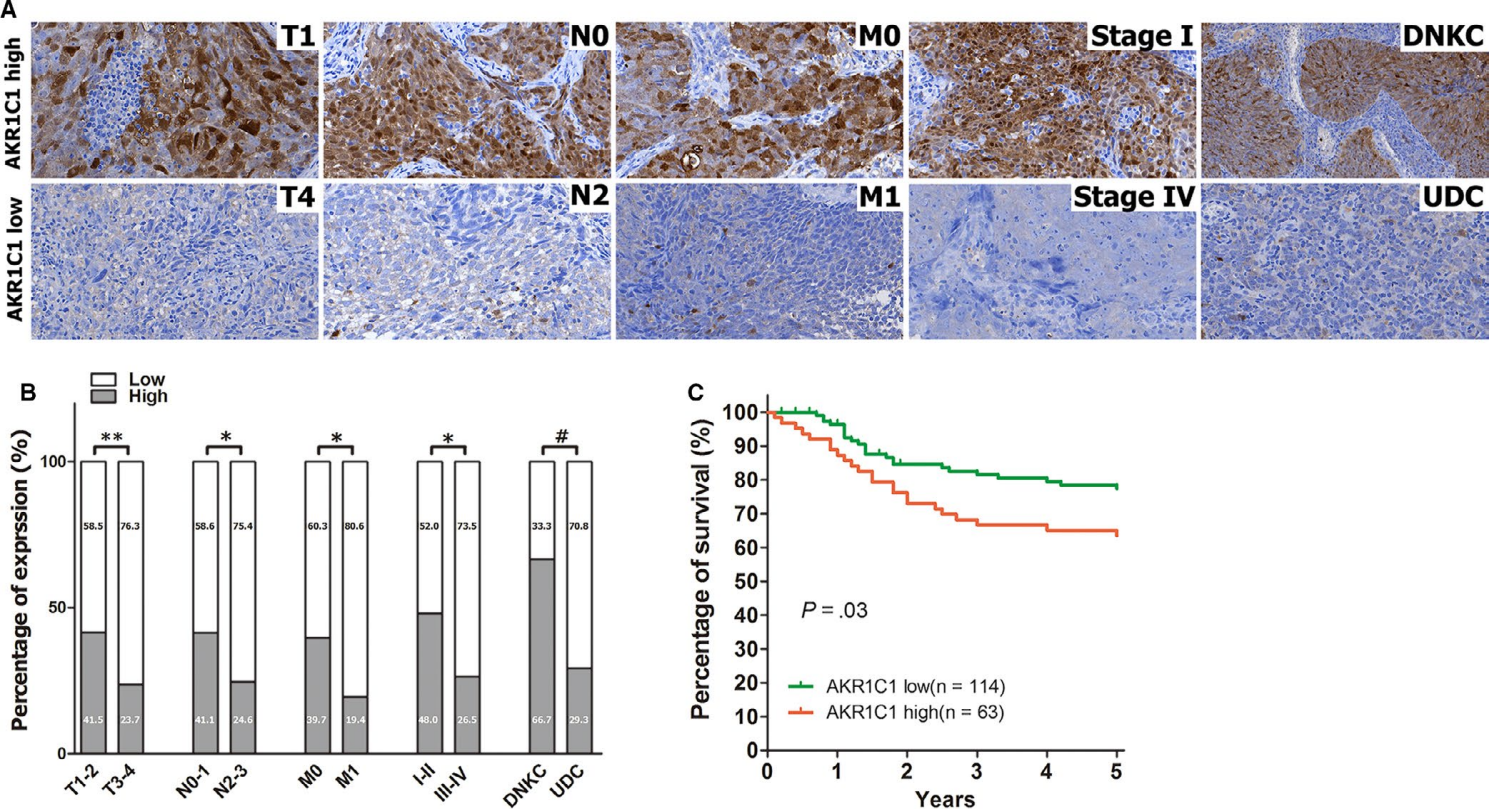
Loss of AKR1C1 was associated with advanced TNM stage while with good prognosis in NPC patients. (A) Representative images of AKR1C1 expression in NPC biopsies with different TNM stages. High expression of AKR1C1 was observed in the T1, N0, M0, and I stage and differentiated histological subtype (DNKC) of NPC biopsies, while low expression of AKR1C1 was detected in the T4, N2, M1, and IV stage and differentiated histological subtype (UDC) of tumours. (B) Numbers and percentages of cases with high or low expression of AKR1C1 according to different clinicopathological features. (C) Low AKR1C1 expression was associated with a good prognosis in NPC patients. *P < .05, **P < .01, ^#^ *P* < .001. Abbreviations: NPC, nasopharyngeal carcinoma; AKR1C1, human 20‐keto reductase family 1 member C1; DNKC, differentiated non‐keratinized squamous carcinoma; UDC, undifferentiated non‐keratinized carcinoma

The relationships between AKR1C1 expression and the clinicopathologic characteristics of NPC patients were summarized in Table 2. No significant association was identified between AKR1C1 expression and the age (*P* = 1.000) or sex (*P* = .755) of 177 NPC patients (Table 2). AKR1C1 expression was, however, significantly associated with the histological type (*P* < .001), tumour size (T classification) (*P* = .030), lymph node metastasis (N classification) (*P* = .040), distant metastasis (M classification) (*P* = .038) and clinical stage (*P* = .005) (Table 2). Low AKR1C1 expression was more frequently observed in undifferentiated non‐keratinized carcinoma (UDC), T3‐T4, N2‐N3, M1 and stage III‐IV tumours than in differentiated non‐keratinized squamous carcinoma (DNKC), T1‐T2, N0‐N1, M0 and stage I‐II tumours (Figure 2A and 2B, Table 2). Although AKR1C1 loss was associated with advanced clinicopathological characters, high AKR1C1 expression was associated with a poor prognosis in NPC patients (Figure 2C). These data implied that AKR1C1 loss may be an accompanied molecular event, while not a drive event in the progression of NPC.

**TABLE 2:** Correlation between the clinicopathological features and AKR1C1 expression in 177 NPC patients

Characteristics	Case No.(n)	AKR1C1 expression		χ^2^	*P*
		High (n,%)	Low (n,%)		
Sex					
Female	46	15 (32.61)	31 (67.39)	0.098	.755
Male	131	48 (36.64)	83 (63.36)		
Age (years)					
<50	82	29 (35.37)	53 (64.63)	0.000	1.000
≥50	95	34 (35.79)	61 (64.21)		
Histological type					
DNKC	30	20 (66.67)	10 (33.33)	13.626	<.001
UDC	147	43 (29.25)	104 (70.75)		
T classification					
T1‐T2	118	49 (41.53)	69 (58.47)	4.686	.030
T3‐T4	59	14 (23.73)	45 (76.27)		
N classification					
N0‐N1	116	48 (41.38)	68 (58.62)	4.210	.040
N2‐N3	61	15 (24.59)	46 (75.41)		
M classification					
M0	141	56 (39.72)	85 (60.28)	4.295	.038
M1	36	7 (19.44)	29 (80.56)		
Clinical stage					
I–II	75	36 (48.00)	39 (52.00)	7.825	.005
III–IV	102	27 (26.47)	75 (73.53)		

Abbreviations: DNKC, differentiated non‐keratinizing carcinoma; M, distant metastasis; N, lymph node metastasis; T, tumour size; UDC, undifferentiated carcinoma.

### 3.3 Knock‐down of AKR1C1 by siRNA showed no impact on cell proliferation, migration and invasion in NPC cells

**Figure 3:**
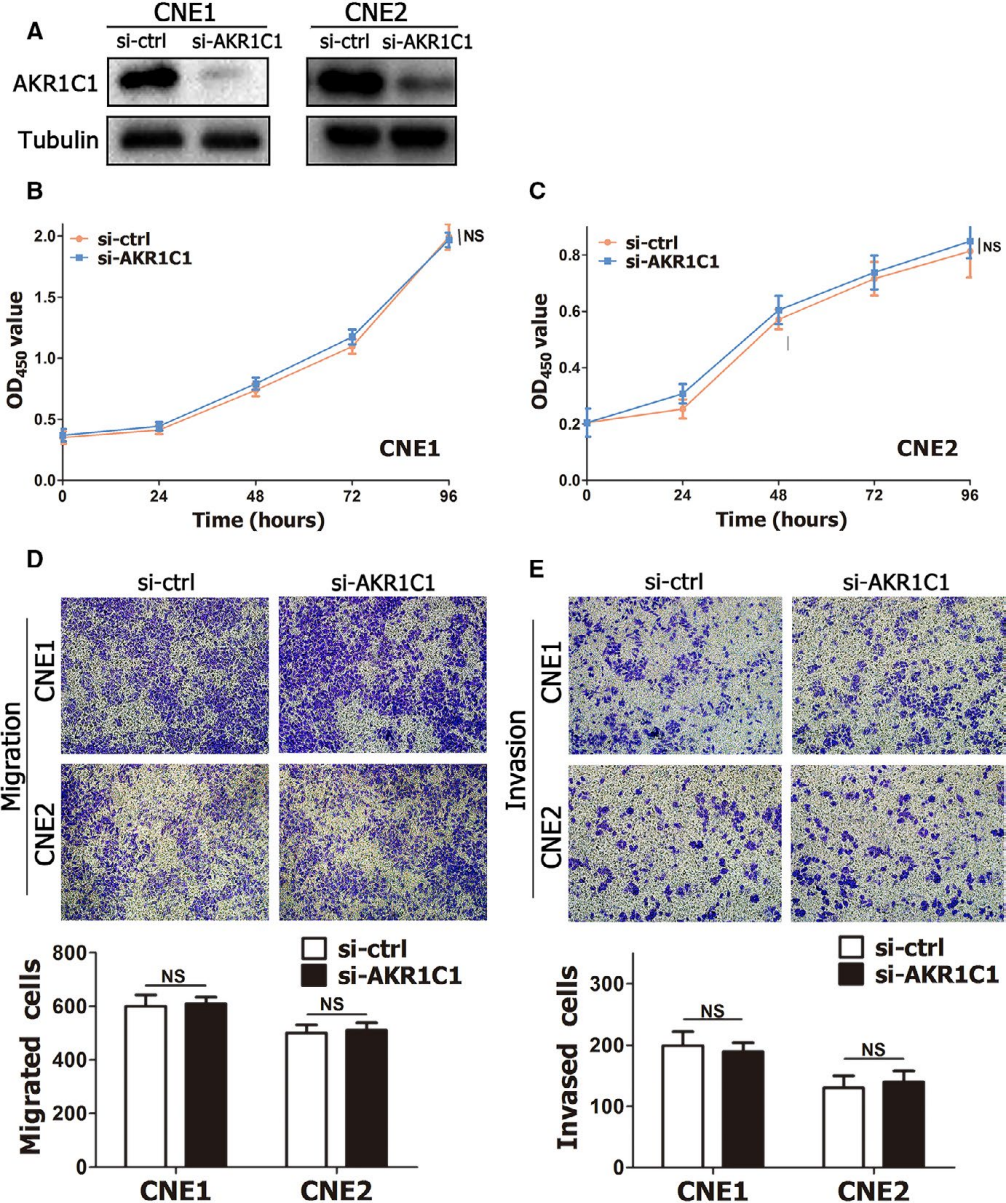
Knock‐down of AKR1C1 by siRNA was not involved in cell proliferation, migration and invasion in NPC cells. (A) Western blot assay showed that AKR1C1 was successfully knocked down by siRNA in CNE1 and CNE2 cells. (B‐C) The effects of AKR1C1 silencing on cell proliferation were measured by CCK8 assay. (D‐E) Migrative and invasive activities of the si‐AKR1C1 and si‐ctrl groups of NPC cells were based on transwell assays. The average number of cells per field was determined from three repeated independent experiments. NS, not significant, *P* > .05. Abbreviations: NPC, nasopharyngeal carcinoma; AKR1C1, human 20‐keto reductase family 1 member C1

To investigate the function of AKR1C1 in NPC cells, CNE1 and CNE2 cells were transiently transfected with siRNA. According to Western blot assay results, AKR1C1 was successfully knocked down by siRNA in NPC cells (Figure 3A). The CCK8 assay showed that AKR1C1 silencing did not promote proliferation of NPC cells (Figure 3B and 3C). In addition, wound healing assays (Figure S2) and transwell assays (Figure 3D and 3E) showed that depleting endogenous AKR1C1 by siRNA did not increase the invasion and migration of NPC cells. These data indicated that AKR1C1 was not directly involved in the malignant biological behaviours of NPC cells.

### 3.4 Knock‐down of AKR1C1 by siRNA enhanced cisplatin‐induced inhibition of cell proliferation and cell cycle arrest

**Figure 4:**
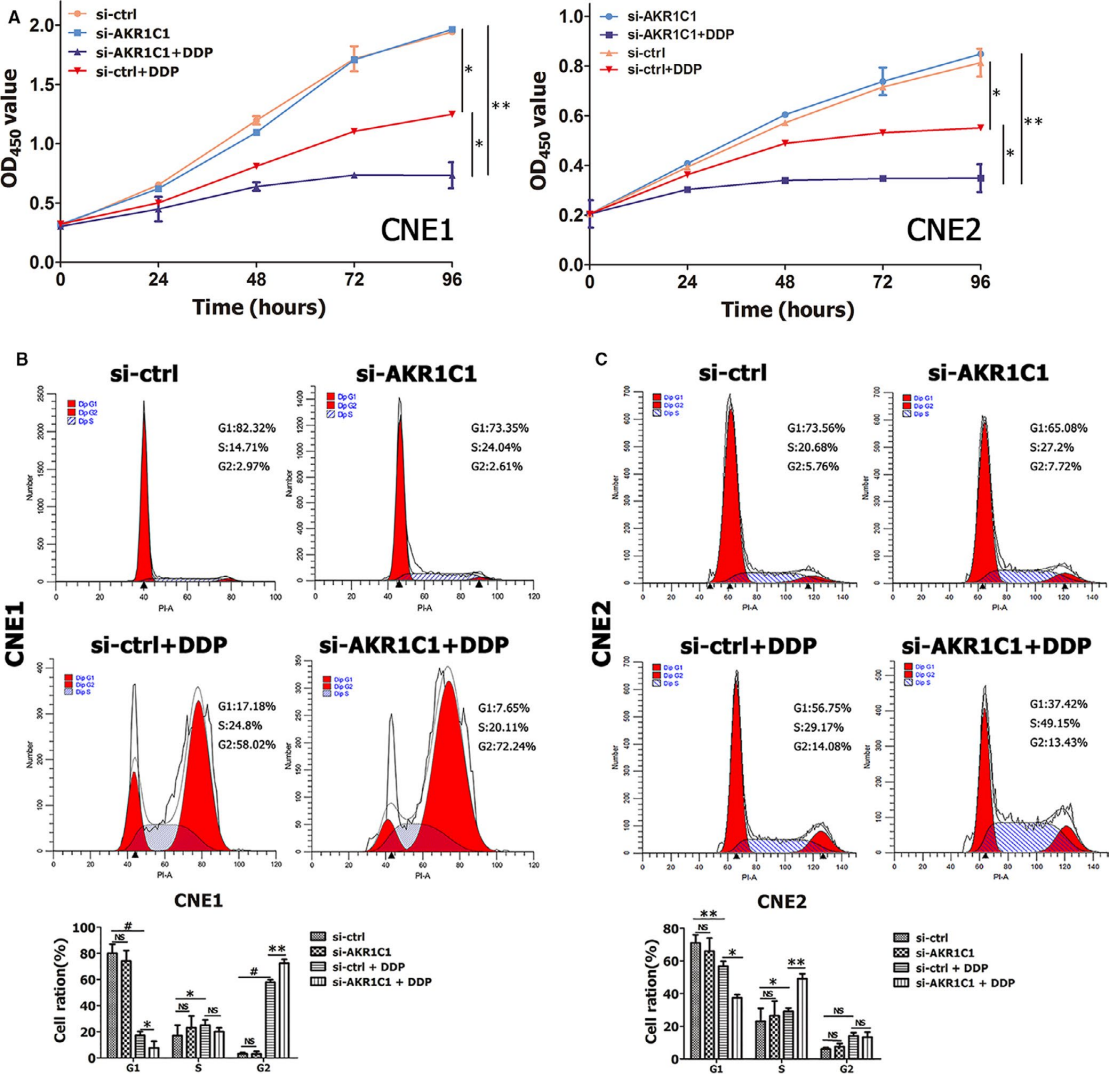
Knock‐down of AKR1C1 by siRNA enhanced cisplatin‐induced inhibition of cell proliferation and arrest of cell cycle. (A) Down‐regulation of AKR1C1 sensitized NPC cells to cisplatin's toxicity on cell proliferation according to CCK8 assays. (B and C) Cisplatin blocked G2/M and G1/S transition in CNE1 and CNE2 cells, respectively. Meanwhile, AKR1C1 silencing sensitized NPC cells to cisplatin's inhibiting effects on cell cycle according to flow cytometry. The average number of cells per field was determined from three repeated independent experiments. *P < .05, **P < .01, ^#^ *P* < .001, NS, not significant, *P* > .05. Abbreviations: AKR1C1, human 20‐keto reductase family 1 member C1; DDP, cisplatin

To determine whether knock‐down of AKR1C1 by siRNA may increase cisplatin sensitivity or not, CNE1 and CNE2 cells were treated with cisplatin for 48 hours after transfected with siRNA. Figure 4A showed that down‐regulation of AKR1C1 sensitized NPC cells to cisplatin's toxicity on cell proliferation. Cisplatin blocked G2/M and G1/S transition in CNE1 and CNE2 cells, respectively. In addition, AKR1C1 silencing sensitized NPC cells to cisplatin‐induced cell cycle arrest (Figure 4B and 4C). These results suggested that AKR1C1 attenuated cisplatin‐induced cytotoxicity and cell cycle arrest in NPC cells.

### 3.5 Knock‐down of AKR1C1 by siRNA increased cisplatin‐induced apoptosis

**Figure 5:**
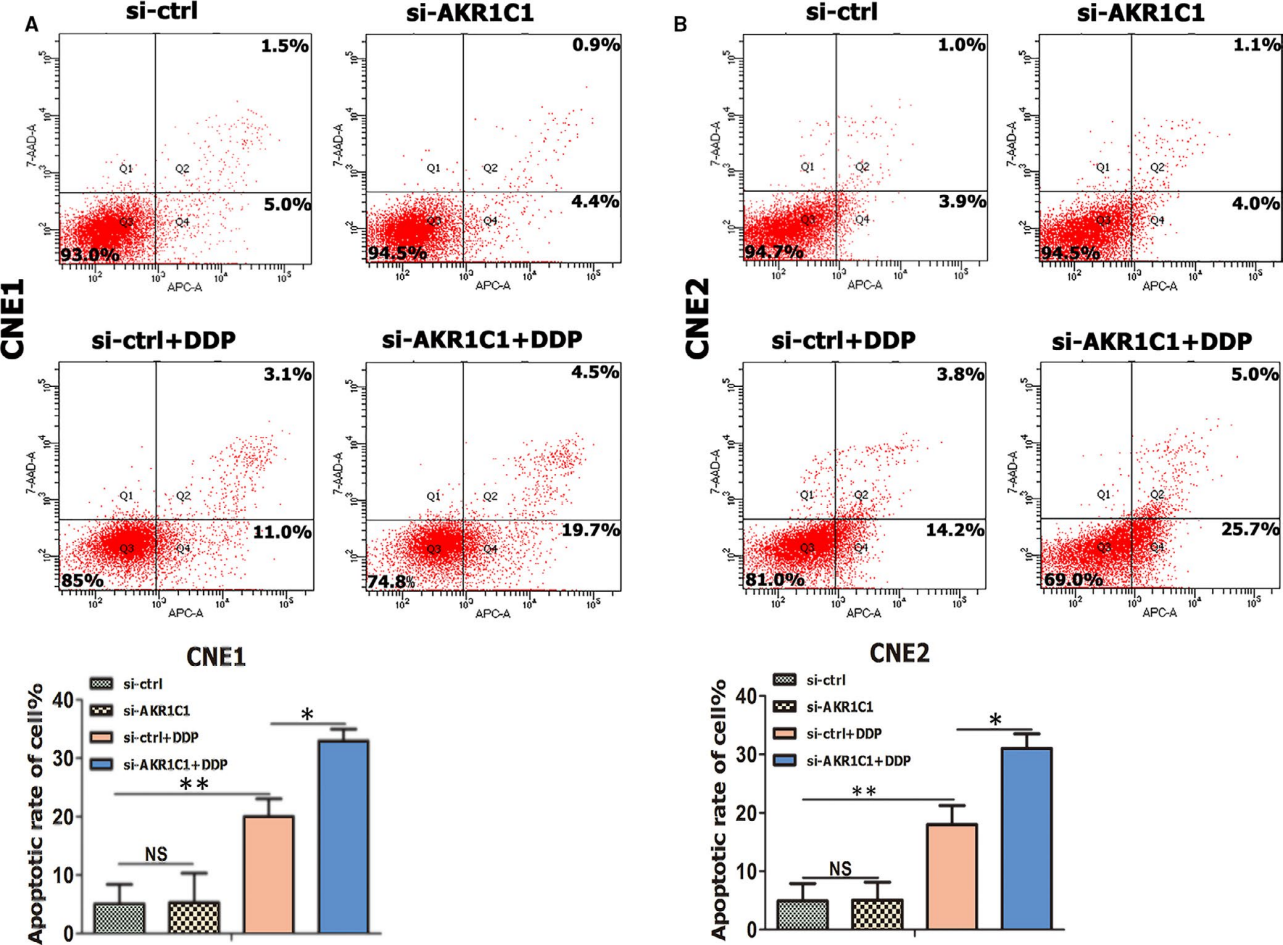
Knock‐down of AKR1C1 by siRNA reversed cisplatin resistance and induced apoptosis in NPC cells. (A and B) After treated with cisplatin for 48 hours, si‐ctrl and si‐AKR1C1 transfected cells were assayed by flow cytometry for calculating apoptotic cells. Results are presented as the mean ± standard error of the mean. The average number of cells per field was determined from three repeated independent experiments.*P < .05, **P < .01; NS, not significant, *P* > .05. Abbreviations: AKR1C1, human 20‐keto reductase family 1 member C1; DDP, cisplatin

To investigate the effect of AKR1C1 knock‐down on cisplatin‐induced cell apoptosis, si‐ctrl and si‐AKR1C1 transfected NPC cells (CNE1 & CNE2) were treated with cisplatin for 48 hours and then assayed by flow cytometry for calculating apoptotic cells (Figure 5A and 5B). Meanwhile, morphology of apoptotic cells’ nuclei was assayed by DAPI staining (Figure S4). Both flow cytometry and nuclear staining showed that AKR1C1 knock‐down increased cisplatin‐induced cell apoptosis. These results suggested that AKR1C1 attenuated cisplatin‐induced apoptosis in NPC cells.

## 4 DISCUSSION

Cisplatin is widely used for treatment of patients with solid tumours such as lung, prostate and cervix carcinoma, but is much less sensitive in colon and breast cancers. Cisplatin is also included in the routine regime of treatment of NPC.^1^ AKR1C1 is reported to be overexpressed in cisplatin‐resistant cancer cell lines^14^, ^16^ and up‐regulated in metastatic lesions of human bladder cancer.^17^ Overexpressed AKR1C1 positively correlates to cisplatin resistance in HNSCC cells,^16^ bladder cancer cells^17^ and colon cancer cells.^14^ In this study, we found AKR1C1 contributing to cisplatin resistance in NPC in vitro. This research establishes AKR1C1 as a potential marker predicting cisplatin responses and a molecular target of NPC treatment.

Most NPC cases are advanced cancer at first diagnosis, and chemotherapy combined with radiotherapy is the standard regime for these patients.^3^, ^4^, ^5^, ^6^ Cisplatin‐based chemotherapy is the first‐line drug for chemoradiotherapy in NPC.^6^ Unfortunately, some NPC patients are cisplatin resistance, which leads to therapeutic failure and increases the risk of tumour relapse or distant metastasis.^18^, ^19^, ^20^ The major factors involved in the mechanism of cisplatin resistance include disrupted membrane transporters for cisplatin uptake or efflux, DNA repair proteins and apoptosis associated proteins or MAPKs.^21^ AKR1C1, a member of the Aldo‐keto reductase superfamily (AKRs),^8^ was reported as a new player in chemotherapy resistance recently.^16^, ^22^ The mechanism of chemotherapy resistance of AKR1C1 included reducing the production of reactive oxygen species, eliminating free radicals and inactivating anticancer drugs, thereby reducing DNA damage and inhibiting apoptosis.^11^, ^12^, ^13^, ^14^, ^23^ Accumulating data showed that AKR1C1 contributed to cisplatin resistance in multiple tumours. Overexpression of AKR1C1 in bladder,^13^ gastric,^17^ ovarian,^24^, ^25^, ^26^ cervical and lung cancer^26^ reduced the sensitivity to cisplatin. AKR1C1 is a poor prognostic factor for recurrence and death of HNSCC patients.^16^

Our data showed that AKR1C1 protein down‐regulated in cancerous tissue when compared with non‐cancerous nasopharyngeal columnar epithelia (Figure 1A and 1B). Lower expression of AKR1C1 was observed in advanced NPC cases with higher grade of T, N, M and clinical stage (Figure 2). Meanwhile, expression level of AKR1C1 in differentiated non‐keratinized carcinoma (DNKC) was higher than in undifferentiated non‐keratinized carcinoma (UDC) (Figure 1A). These data implied that AKR1C1 gradually lost in the progression processes of NPC. However, expression data in immortalized nasopharyngeal epithelial cells and NPC cells showed that AKR1C1 lost in 3 immortalized nasopharyngeal epithelia cells (Figure 1C). The underlining mechanisms contributing to the difference of AKR1C1 expression between nasopharyngeal epithelial tissue and immortalized nasopharyngeal epithelial cell lines are largely unknown. Interestingly, IHC assays showed that AKR1C1 also lost in metaplastic squamous epithelia of nasopharyngeal tissues (Figure S1). It is well known that pseudostratified columnar epithelial cells, which lining the surface of nasopharynges, are prone to underwent columnar‐to‐squamous metaplasia when suffered various kinds of stimulation. Primary epithelial cells were genetically manipulated to produce immortalized cells. Metaplasia could happen due to various kinds of stimulation in this process of culture in vitro. Whether squamous metaplasia and AKR1C1 loss happen in immortalized nasopharyngeal epithelial or not still need to be identified. Whatever, AKR1C1 lost in several NPC cells lines (Figure 1C). Taken together, loss of AKR1C1 in NPC tissues and NPC cells implied that AKR1C1 down‐regulation was a key molecular event in the development and progression of NPC.

Although AKR1C1 lost in NPC tissues and several NPC cells lines, loss of AKR1C1 is a good prognostic factor in NPC patients (Figure 2C). These seemingly contradictory results might be derived from the distinctive function of AKR1C1 in NPC. Our data showed AKR1C1 silencing by siRNA had no impact on cell proliferation, migration and invasion (Figure 3 and Figure S2),while AKR1C1 silencing sensitized NPC cells to the cytotoxicity of cisplatin (Figures 4, 5 and Figure S4). These data implied that AKR1C1 was not a driver factor in the process of carcinogenesis and progression in NPC, AKR1C1 loss maybe an accompanying molecular event which contributed only to the chemotherapeutic sensitivity to cisplatin while not the malignant biological behaviours of NPC. Although AKR1C1 is reported to be involved in tumour metastasis through interaction with STAT3 in NSCLC^27^ and to promote invasion of bladder cancer cells, evidence supporting the facilitation of metastasis is still lacking.^17^ Taken together, the functional explorations of AKR1C1 provided a reasonable answer for the contradictory phenomenon between the expression data and the prognostic data in NPC.

Actually, although therapeutic methods improved greatly in recent years, histological subtype remains a prognostic factor determining the long‐term survival outcome of patients with NPC.^28^, ^29^, ^30^ Moreover, the non‐keratinized carcinoma subtype has the best prognosis, while the keratinized squamous cell carcinoma subtype has the worst prognosis.^30^ However, there is not a consensual therapeutic protocol for the different specific histological subtypes of NPC so far.^31^ Our data showed that AKR1C1 more frequently lost in undifferentiated non‐keratinized carcinoma than in differentiated non‐keratinized carcinoma. Distinct expression profile of genes including chemoradiotherapy‐associated genes such as AKR1C1 may result in different prognosis in different subtypes of NPC. Moreover, because of no personalized therapeutic regime existed between undifferentiated non‐keratinized carcinoma and differentiated non‐keratinized carcinoma, the prognostic and chemotherapeutic reactive differences were not documented in the published literatures so far. Our data implied that AKR1C1 may contribute to different chemotherapeutic sensitivity between differentiated and undifferentiated histological subtype of NPC.

In summary, our study demonstrates that loss of AKR1C1 could enhance cisplatin sensitivity in NPC cells. Increased chemotherapeutic sensitivity corroborated AKR1C1 loss as a good prognostic factor in NPC patients.

## 5 CONCLUSIONS

AKR1C1 loss is identified as a good prognostic factor in the advanced staged cases of NPC in this study. Cisplatin sensitivity increased in AKR1C1‐silenced NPC cells warrants further validating AKR1C1 as a potential marker for predicting cisplatin response in NPC patients. New therapeutic strategies must take into consideration when managing different histological subtype and molecular subtype of NPC with differentially expressed chemotherapy‐associated genes such as AKR1C1.

## CONFLICT OF INTEREST

The authors report no conflicts of interest in this work.

## AUTHOR CONTRIBUTION

Shengjun Xiao and Xiaoling Zhang designed the study, offered important suggestion to manuscript writing and did manuscript final approval. Chen Zhou, Guowen Shen, Fan Yang, Jingling Duan, Zhen Wu, Mingqing Yang, Yi Liu and Xueli Du performed in human tissue samples and in vitro experiments. Chen Zhou performed data analysis and wrote the paper.

## Supporting information

Supplementary Material

## Data Availability

All data that support the findings of this study are available from the corresponding author upon reasonable request.
