# Supplementary material for: Loss of AKR1C1 is a good prognostic factor in advanced NPC cases and increases chemosensitivity to cisplatin in NPC cells
Source: J Cell Mol Med. 2020 Apr 19;24(11):6438–47. doi: 10.1111/jcmm.15291 (PMC7294127; doi:10.1111/jcmm.15291)
Supplement: Supplementary file 1 — Supplementary Material [file JCMM-24-6438-s001.docx]

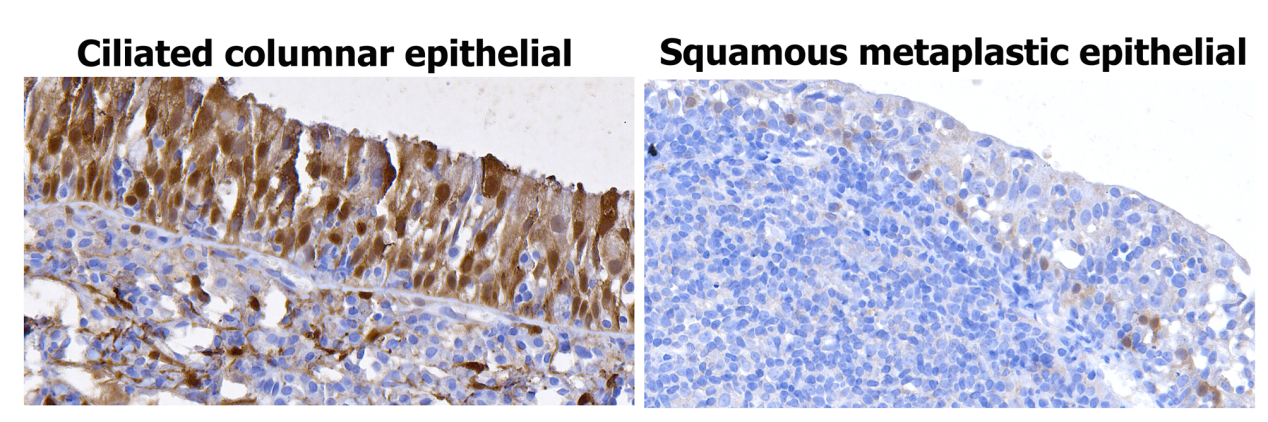


Figure S1 High expression of AKR1C1 in ciliated columnar epithelial and AKR1C1 knockdown in metaplastic epithelial .

Abbreviations: AKR1C1, Human 20-keto reductase family 1 member C1.


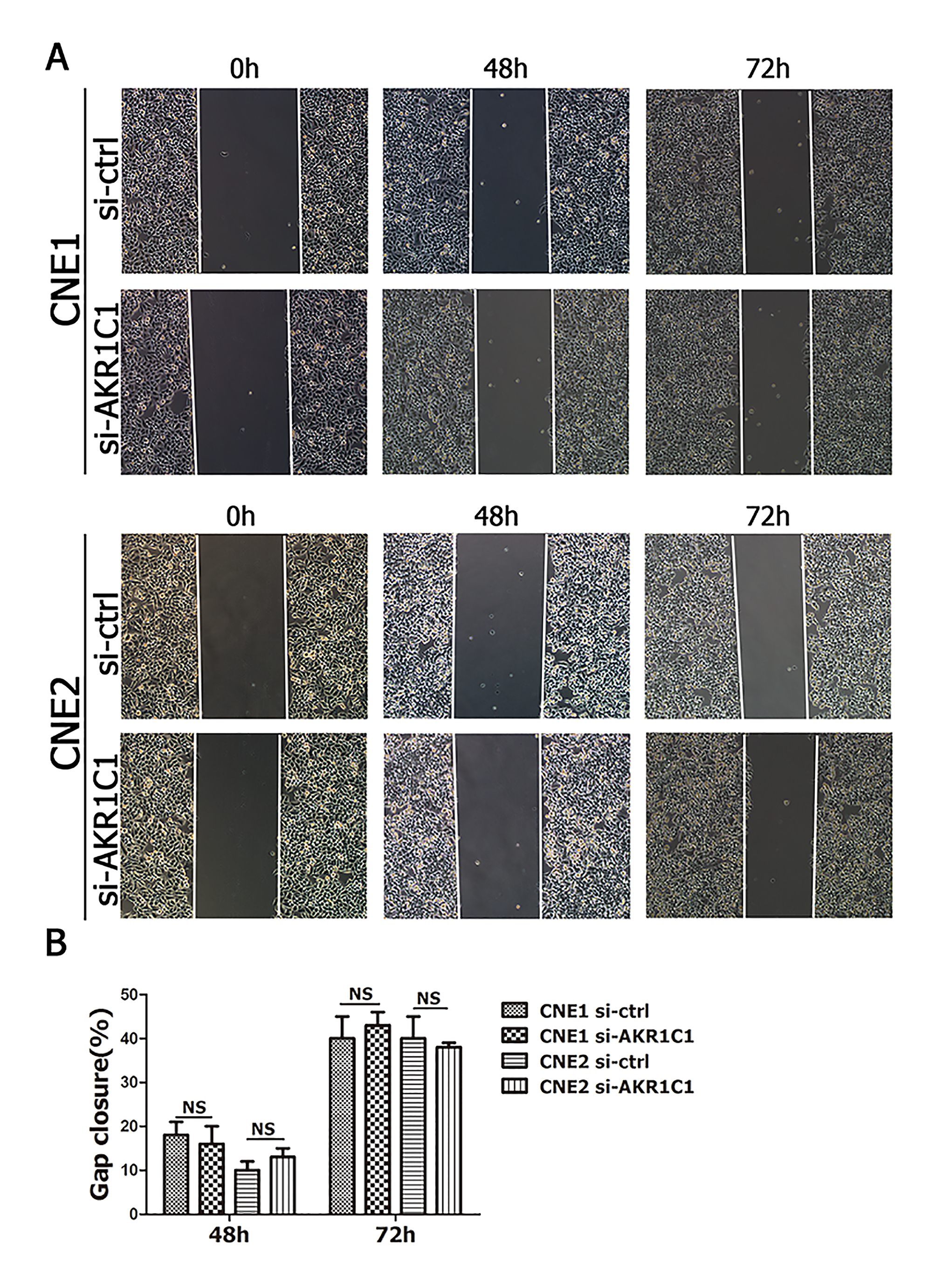


Figure S2 AKR1C1 knockdown showed no affect on cell migration in NPC cells by wound scratch assays. NS, not significant, *P*>0.05.

Abbreviations: AKR1C1, Human 20-keto reductase family 1 member C1.


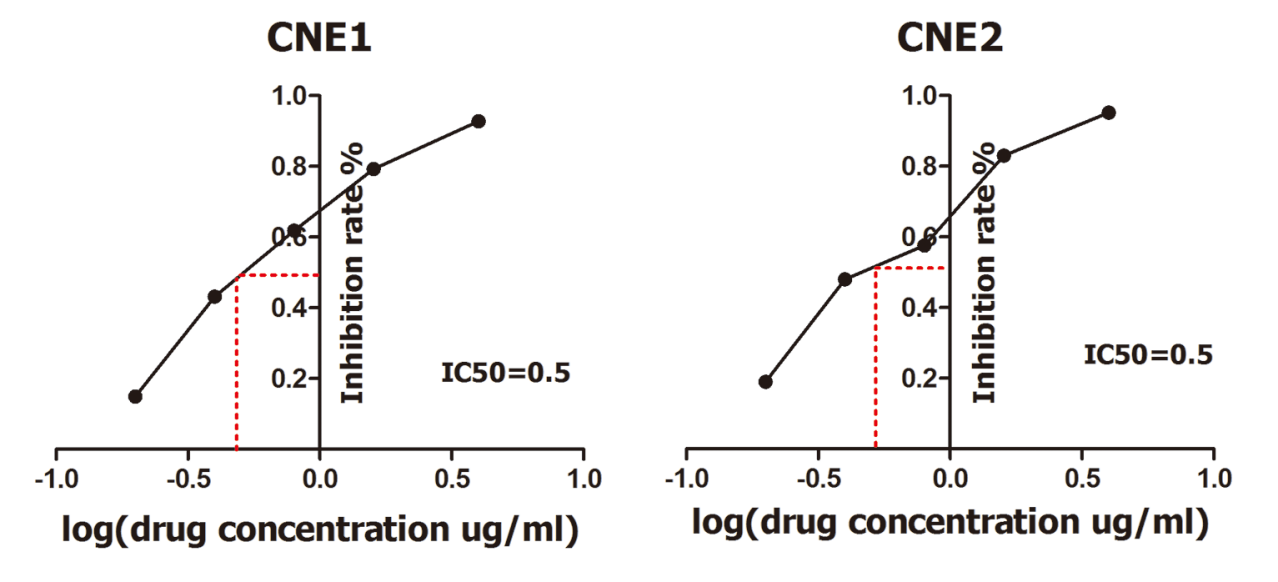


Figure S3 After treated with 0.5 μg/mL cisplatin for 48 h, CNE1 and CNE2 cells achieved half inhibition.

Abbreviations: AKR1C1, Human 20-keto reductase family 1 member C1; IC50, half-maximal inhibitory concentration.


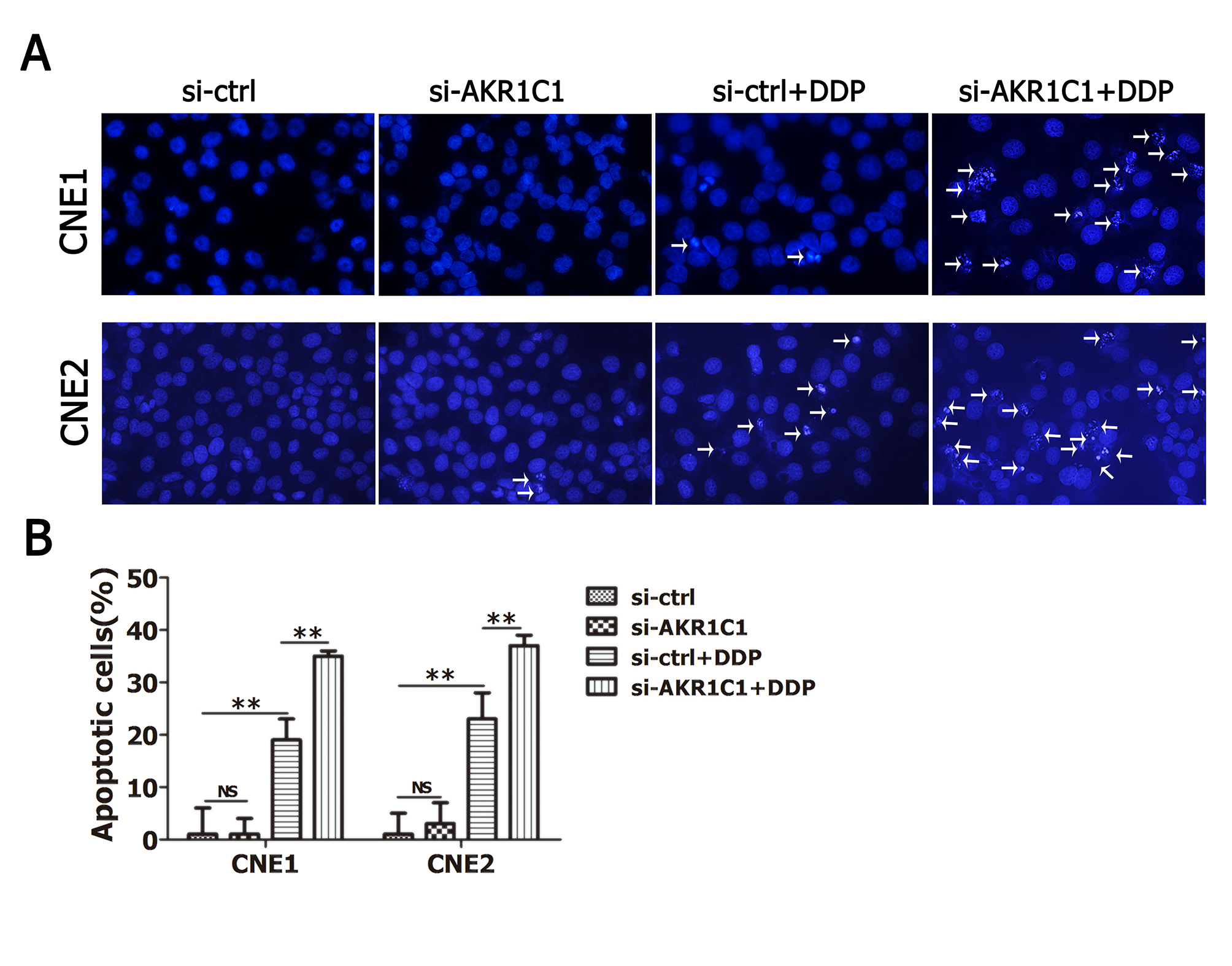


Figure S4 Knockdown of AKR1C1 by siRNA reversed cisplatin resistance and induced apoptosis in NPC cells. (A) Si-ctrl and si-AKR1C1‑transfected cells were treated with cisplatin, and after 48 h of treatment, cell apoptosis was determined by DAPI staining. Images showed cell nuclei in blue (DAPI). (B) Results are presented as the mean±standard error of the mean. ***P*<0.01; NS, not significant, *P*>0.05.

Abbreviations: AKR1C1, Human 20-keto reductase family 1 member C1; DDP, Cisplatin; Arrow Head: Apoptoic cell.
